# Population norms for the EQ-5D-3L and EQ-5D-5L in Romania

**DOI:** 10.1186/s12955-023-02144-8

**Published:** 2023-07-29

**Authors:** Elena Olariu, Raluca Caplescu, Luke Vale, Ileana Gabriela Niculescu-Aron, Yemi Oluboyede, Marian Sorin Paveliu

**Affiliations:** 1 Health Economics Group, Population Health Sciences Institute, Newcastle University, Newcastle Upon Tyne, UK; 2 Department of Statistics and Econometrics, Bucharest University of Economic Studies, Bucharest, 010374 Romania; 3 Department of Pharmacology, Titu Maiorescu University, Bucharest, 031593 Romania; 4 Romanian Academic Society, Bucharest, 020071 Romania

**Keywords:** EQ-5D-3L, EQ-5D-5L, Romania, Health-related quality of life, Population norms

## Abstract

**Background:**

The majority of patient reported outcome measures (PROMs) don’t have population norms in Romania. This is the case with the EQ-5D as well. Therefore, we aimed to estimate population norms for the Romanian versions of the EQ-5D-5L, EQ-5D-3L, their indexes, and the EQ-VAS.

**Methods:**

A cross-sectional survey was conducted in all regions of Romania from November 2018 to November 2019. A three-stage probability sampling procedure stratified by region and settlement size was used to select a representative sample. Interviews were computer-assisted and conducted in respondents’ homes by trained interviewers. Health status was assessed with the EQ-5D-5L, the EQ-5D-3L and the EQ VAS. Descriptive statistics were used to estimate population norms by age groups and sex for the EQ-5D-5L, the EQ-5D-3L, their indexes and the EQ VAS. Population norms were weighted using survey weights. Indexes for the EQ-5D questionnaires were estimated using the recently developed Romanian value sets.

**Results:**

Data from 1,649 interviews was analysed in the present study. Survey weights were used so that sex and place of residence ratios for the weighted sample matched the Romanian general population distribution. Participants’ mean age was 47.4 years (SE = 1.157) and 50.3% of them reported being in good health. The dimension for which people reported the highest number of problems for both questionnaires was the pain/discomfort dimension. Men aged 35 plus reported fewer problems with pain/discomfort than women for both the EQ-5D-5L and EQ-5D-3L. Health decreased with age as shown by the decrease from age group 18–24 to age group 75 plus in the indexes of both questionnaires: from 0.977 (SE = 0.005) to 0.765 (SE = 0.017) for EQ-5D-5L and from 0.981 (SE = 0.005) to 0.784 (SE = 0.019) for EQ-5D-3L. There was 29.9 points drop in the EQ VAS score between the youngest and oldest group.

**Conclusions:**

Population norms for the Romanian versions of the EQ-5D-5L, EQ-5D-3L, their indexes, and the EQ VAS are now available. These can now be used as reference values by healthcare professionals, researchers and decision-makers leading to a further development of health-related quality of life research in Romania.

**Supplementary Information:**

The online version contains supplementary material available at 10.1186/s12955-023-02144-8.

## Background

Nowadays researchers and healthcare professionals have at their disposal several methods to determine an individual’s health status. These methods range from reports that come from people with recognized professional training in the assessment made (clinician-reported outcome measures) to reports that come directly from that respective individual (patient-reported outcome measures—PROMS) or from people who can report on their behalf but with no relevant training in the assessment made (observer-reported outcome measures) [1]. Of these, the use of PROMS in research, health service evaluation and clinical practice has steadily increased over the years [2]. They currently cover a broad range of health-related concepts, such as health-related quality of life (HRQoL), functional status, symptoms and symptom burden, health behaviours, and the patient’s health care experience [3].

PROMS that measure HRQoL are the most commonly used type of PROMS [4]. These can be specific to certain conditions, symptoms, interventions or treatments or generic, designed to be used across diseases [5]. Some of the most widely used generic instruments to measure HRQoL are the SF-36 [6] and the EQ-5D [7]. The EQ-5D is a simple to use instrument developed by the EuroQoL Group that consists of a descriptive system with five dimensions (mobility, self-care, usual activities, pain/discomfort and anxiety/depression) and a visual analogue scale (VAS) [8]. Currently two versions of the questionnaire are available, the EQ-5D-3L and EQ-5D-5L, with the latter having improved psychometric properties [9, 10]. In Romania, the EQ-5D has mainly been used in studies on different patient populations [11] and value sets for both EQ-5D-3L and 5L have recently been developed [12, 13].

One attractive feature of HRQoL PROMS, including the EQ-5D, is that they can be used to monitor population health status over time [14] or to assess the impact of public health interventions [14], treatments [4] or healthcare policies in a given population [15]. More exactly, they can be used to estimate the burden of certain diseases or to evaluate certain types of care [16] by comparing the health of a specific patient group or of a group of patients that uses a certain type of care with that of the general population. Additionally, they can be useful in determining the effects of those health interventions for which control groups do not exist [17] or in identifying inequalities or policy gaps within a country. Finally, they can be useful in regional and cross-country comparisons. All these can be determined if population norms for PROMS that measure HRQoL are available. To date, in Romania, there is a scarcity of population norms for PROMS that measure HRQoL and, to the best of our knowledge, population norms have only been developed for SF-36 [18]. Given that the EQ-5D is the preferred instrument included in the Romanian health technology assessment guidelines [19], the objective of this study was to estimate population norms for the Romanian version of both EQ-5D-3L and EQ-5D-5L.

## Methods

### Study design and sample

We used data from an Omnibus survey that was conducted in all regions of Romania from November 2018 to November 2019. The objective of this survey was to provide HRQoL data to support health technology assessment and reimbursement decisions in Romania by developing value sets and population norms for EQ-5D-3L and EQ-5D-5L. The survey was based on a three-stage probability sampling procedure stratified by region and settlement size that led to the selection of the 32 settlements where interviews took place. Households were selected using a random walk procedure [20] that had as starting point the address of different polling stations that were randomly selected for each settlement. Individuals within households were selected using the next birthday rule [21]. Only adults older than 18 who were residing in Romania at the time of the survey were invited to take part in the study. The sample size was estimated at 1,613 respondents with a maximum error of ± 3% at a confidence level of 95%. More details on the sampling procedure can be found elsewhere [22].

Interviews were computer-assisted, face-to-face and took place in respondents’ homes. They were performed by interviewers that were trained by the local study team using standardized training materials in two face-to-face training sessions in October 2018 and June 2019. Interviewers were selected from members of patients’ associations and health surveyors from the National Authority of Quality Management in Health from Romania.

### Questionnaire

The interview was standardized in accordance with the latest interviewer guidelines approved by the EuroQoL Group. The software used to collect the data was developed by the EuroQoL Group(EQ-VT v2.1) to support EQ-5D-5L valuation studies. The interview comprised of five-block sections. Section one consisted of some background questions, the EQ-5D-5L questionnaire and the EQ VAS. Section two included warmup exercises and valuation tasks for the EQ-5D-5L and EQ-5D-3L. Section three consisted of discrete choice experiments for EQ-5D-5L and section four included the EQ-5D-3L questionnaire and the EQ VAS. The last section of the interview consisted of sociodemographic questions on residence area, ethnicity, caregiver and parenting status, health literacy, preference over length or quality of life, marital status, education level, religion (affiliation, general religiosity, participation in religious services, praying), employment status and income. This structure of the interview has been used before in other valuation studies [23, 24]. In this paper we only analysed data from section one, four and five of the interview.

### Data quality control

A random 61.5% of the sample was contacted by telephone to check that the interview took place by confirming the settlement, respondent’s age and the approximate duration of the interview. Sections one, two, three and four of the interview had a hard choice format, meaning that the interviewer could not have proceeded to the next question unless an answer was provided to the current question. Sections two and three of the interview were subject to an additional data quality control check that has been described elsewhere [13, 22].

### Statistical analysis

All statistical analyses were performed with STATA version 18. Frequencies and percentages were used to describe categorical variables and measures of central tendency and dispersion were used for continuous variables.

Respondents’ answers to EQ-5D-3L and 5L were converted into index values using the recently developed value sets for Romania for the two questionnaires [12, 13]. Descriptive statistics such as percentages and standard errors were calculated for each level in all five dimensions for both questionnaires for the whole sample and stratified by age groups, sex and place of residence. In line with the recommendations of the EuroQoL Group for estimating population norms [25], the following age groups were used: 18–24, 25–34, 35–44, 45–54, 55–64, 65–74 and 75 + years. The variable place of residence was used as a variable with two categories: rural and urban.

For the index values and the EQ VAS, descriptive statistics, such as the mean, standard deviation, percentile 25, median and percentile 75, were calculated for the whole sample and by age and sex for both questionnaires. We also reported the mean EQ VAS and index values for the most frequently reported health states for both questionnaires. Finally, for both questionnaires, we estimated the percentage of people reporting having no problems in all five dimensions of the questionnaires stratified by age groups.

Design weights, non-response weights and poststratification weights were included in the estimation of the final survey weights that were used in all analyses. We used survey weights to bring our sample back to being representative of the Romanian general population on a set of sociodemographic characteristics, such as age, sex and place of residence. This is in line with the current consensus in the survey literature that recommends the use of survey weights for descriptive statistics [26].

We calculated design weights as the inverse of the probability of being selected into the sample at each stage and non-response weights as the inverse of the probability of response for each primary sample unit. To account for any potential differences between our sample and the Romanian general population in terms of age, sex and place of residence, post-stratification weights were generated using a raking procedure. Population control totals for the raking procedure were taken from the 2011 Romanian census for the following variables: age, sex and place of residence.

In order to account for all elements of our complex design (weighting, stratification and clustering) we had, first of all, to correct our sample’s results with survey weights and then to use bootstrapping to estimate the associated sampling errors.

We also qualitatively compared, without performing any formal statistical analysis, the Romanian EQ-5D-5L and EQ-5D-3L population norms with the existing (at the time of writing this manuscript) population norms for other Central and Eastern European countries.

## Results

One thousand six hundred seventy-four interviews were conducted from November 2018 to November 2019. Of these, only 1,649 were used in the analysis: 25 interviews were excluded as they had been performed by an interviewer that was excluded from the interviewers’ team due to being non-compliant with the study’s protocol. Interviews lasted for approximately 47 min (SD = 24).

In the telephone checks performed for quality purposes, contact with respondents was made in 87% of the called phone numbers. Of the people who answered the call, 90.8% confirmed the interview, 4.1% refused to answer any further questions, and 5% did not confirm the interview either because they did not recall having participated in the interview (1.1%) or because the call was answered by somebody different from the respondent that could not confirm whether or not the interview took place (3.9%).

There was no missing data in the main outcome variables (the five dimensions of both questionnaires, the EQ VAS and EQ-5D indexes), the variables used for stratification (age, sex, place of residence) or the variable experience with illness. The only variables that had missing data were marital status, education, work status and income for which missing values varied from 0.5% (education) to 8.1% (income). No missing values were imputed for the purposes of the present manuscript.

The mean age of the included sample was 47.4 years SE = 1.157 (unweighted sample: 48.4 years SD = 16.3). At the time of the survey, the majority of people were employed (53.5%) and most of them were married or living with a partner (61.7%). Our weighted sample overrepresented people with higher education: we had almost two times more highly educated people than the average national statistics (30.1% vs 15.9%). On the other hand, people with average income were underrepresented when compared with the average national statistics (18.5% vs 30.7%) (Table 1).

**Table 1:** Sociodemographic characteristics of the analysed sample

**Variable**		**n (raw count)**	**n (raw %)**	**Romanian general population (%)**	**Weighted sample (%)**	**Standard error**
**Gender**	Women	1072	65	52	52	2.240
**Age**	18–24	139	8.4	9	10.8	2.181
	25–34	199	12.1	15.4	17.7	2.357
	35–44	376	22.8	18.2	20.2	2.196
	45–54	374	22.7	18.9	14.8	1.076
	55–64	244	14.8	15.1	16.6	1.544
	65–74	204	12.4	13.4	11	1.51
	75 or more	113	6.9	9.9	9	1.414
**Residence area**	Urban	1212	73.5	55.2	54.3	2.089
**Marital status**	Single	250	15.2	24.1^a^	21.1	2.506
	Married/living with a partner	1083	65.7	59.1^a^	61.7	2.137
	Divorced/Separated	117	7.1	5.2^a^	6.0	0.842
	Widowed	179	10.9	11.6^a^	11.2	1.137
**Level of education**	No formal education	7	0.4	2	0.8	0.366
	Low	192	11.6	36.9	15.5	2.872
	Medium	830	50.3	45.2	53.5	3.094
	High	611	37.1	15.9	30.1	2.687
**Work status**	Employed	973	59	52.1	53.5	3.259
	Unemployed	35	2.1	3.9	3.4	0.982
	In education	92	5.6	4.8	6.8	1.545
	Retired	415	25.2	26.2	27.3	2.596
	Stay at home/Domestic	117	7.1	7.1	9.1	1.896
**Income**	Above the average	513	31.1	27.9	29.8	3.192
	Average	298	18.1	30.7	18.5	2.102
	Below the average	705	42.8	41.4	51.6	3.427
**Experience with serious illness**	In self	327	19.8	N/A	21.0	2.733
	In family	763	46.3	N/A	44.9	2.363
	In caring for others	270	16.4	N/A	14.6	2.462

*N/A* Not Applicable

^a^General population estimations are based on the legal marital status

**Fig. 1:**
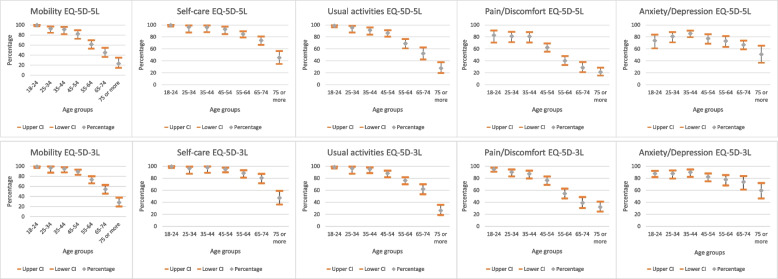
Percentage of people reporting no problems in each dimension of EQ-5D-5L and EQ-5D-3L, respectively, by age group. *CI*, confidence interval

More than 90% of the people reported having no problems with mobility and usual activities up to the age group 35–44 (see Fig. 1). As age increased the percentage of people reporting no problems in those dimensions decreased gradually. For the pain/discomfort dimension, a drop of approximately 22 percentage points can be observed between the percentages of people reporting no problems for the age groups 45–54 and 55–64 for both questionnaires. The highest number of age groups that reported having no problems in percentages higher than 90% was registered for the self-care dimension. In the anxiety/depression dimension, the percentage of people reporting no problems gradually increased up to the age group 35–44 and then decreased again with age. For the age group 18–24, the anxiety/depression dimension was the only one for which the percentage of people reporting no problems was lower than 90% for both questionnaires. In men, the smallest gap between the youngest and the oldest age group that reported having no problems was for the anxiety/depression dimension for both questionnaires. A similar pattern was observed in women. The highest gap between the youngest and the oldest age group that reported having no problems was mobility in men and usual activities in women for both questionnaires (see Supplementary Tables 3, 4, 5 and 6 from Supplementary material). Finally, across all dimensions of both EQ-5D-5L and EQ-5D-3L, the percentage of people from rural areas who reported having no problems was lower than the percentage of people from urban areas that reported the same (see Supplementary Table 7 from Supplementary material).

The most frequently reported health states were 11111 and 11121 for both questionnaires. 61.2% of the people reported being in full health when they filled in the EQ-5D-3L and 50.3% when they filled in the EQ-5D-5L. There was a difference of 42.1 percentage points between the most reported health state and the second most reported health state for EQ-5D-5L and 54.6 percentage points for EQ-5D-3L (see Supplementary Tables 8 and 9 from Supplementary material).

Age group 18–24 reported the best health according to both EQ-5D-5L and EQ-5D-3L indexes (see Table 2, all). A decrease in self-reported health was observed for both questionnaires from age 35 onward. Men reported worse health than women for age groups 25–34 and 45–54 for both questionnaires. The highest difference in self-reported health between sexes was registered for the age group 75 plus for both questionnaires with women reporting poorer health status than men.

**Table 2:** Population norms for the EQ-5D-5L Index and the EQ-5D-3L Index, respectively, for all, men and women, by age groups (weighted sample)

Age group	Indicator	EQ-5D-5L			EQ-5D-3L		
		**All**	**Men**	**Women**	**All**	**Men**	**Women**
**18–24**	**Mean (SE)**	0.977 (0.005)	0.974 (0.009)	0.981 (0.008)	0.981 (0.005)	0.984 (0.006)	0.976 (0.007)
	**95%CI**	0.966—0.987	0.956—0.992	0.966- 0.996	0.971—0.990	0.971—0.996	0.963—0.989
	**P 25**	0.962	0.962	1.000	1.000	1.000	1.000
	**Median**	1.000	1.000	1.000	1.000	1.000	1.000
	**P 75**	1.000	1.000	1.000	1.000	1.000	1.000
**25–34**	**Mean (SE)**	0.963 (0.014)	0.951 (0.027)	0.974 (0.009)	0.976 (0.007)	0.973 (0.013)	0.979 (0.007)
	**95%CI**	0.936—0.991	0.897—1.005	0.958—0.991	0.962—0.991	0.947—0.999	0.966—0.993
	**P 25**	0.962	0.947	0.962	1.000	1.000	1.000
	**Median**	1.000	1.000	1.000	1.000	1.000	1.000
	**P 75**	1.000	1.000	1.000	1.000	1.000	1.000
**35–44**	**Mean (SE)**	0.971 (0.009)	0.979 (0.007)	0.963 (0.012)	0.969 (0.008)	0.968 (0.011)	0.969 (0.007)
	**95%CI**	0.955—0.988	0.966—0.992	0.940—0.987	0.953—0.984	0.947—0.990	0.956—0.983
	**P 25**	0.962	1.000	0.947	1.000	1.000	1.000
	**Median**	1.000	1.000	1.000	1.000	1.000	1.000
	**P 75**	1.000	1.000	1.000	1.000	1.000	1.000
**45–54**	**Mean (SE)**	0.948 (0.008)	0.947 (0.009)	0.950 (0.009)	0.948 (0.008)	0.946 (0.009)	0.951 (0.009)
	**95%CI**	0.933—0.964	0.930—0.964	0.933—0.967	0.932—0.964	0.928—0.964	0.934—0.968
	**P 25**	0.909	0.941	0.909	0.896	0.896	0.896
	**Median**	1.000	1.000	1.000	1.000	1.000	1.000
	**P 75**	1.000	1.000	1.000	1.000	1.000	1.000
**55–64**	**Mean (SE)**	0.905(0.008)	0.915 (0.012)	0.896 (0.010)	0.906 (0.009)	0.907 (0.016)	0.906 (0.009)
	**95%CI**	0.890—0.920	0.892—0.937	0.877—0.915	0.888—0.924	0.876—0.938	0.888—0.924
	**P 25**	0.860	0.867	0.843	0.842	0.852	0.842
	**Median**	0.922	0.947	0.909	0.896	0.914	0.896
	**P 75**	1.000	1.000	0.962	1.000	1.000	1.000
**65–74**	**Mean (SE)**	0.851 (0.017)	0.885 (0.014)	0.826 (0.021)	0.862 (0.015)	0.893 (0.015)	0.838 (0.018)
	**95%CI**	0.817—0.885	0.857—0.913	0.784—0.868	0.832—0.892	0.864—0.922	0.802—0.874
	**P 25**	0.783	0.849	0.744	0.798	0.814	0.774
	**Median**	0.883	0.899	0.869	0.883	0.896	0.858
	**P 75**	0.947	0.961	0.947	1.000	1.000	0.914
**75 or more**	**Mean (SE)**	0.765 (0.017)	0.804 (0.018)	0.743 (0.023)	0.784 (0.019)	0.823 (0.015)	0.760 (0.028)
	**95%CI**	0.732—0.799	0.768—0.839	0.697—0.788	0.747—0.820	0.794—0.852	0.706—0.815
	**P 25**	0.683	0.751	0.651	0.720	0.720	0.720
	**Median**	0.786	0.822	0.759	0.798	0.842	0.774
	**P 75**	0.874	0.887	0.863	0.886	0.890	0.870

*CI* Confidence interval, *P 25* 25^th^ percentile, *P 75* 75^th^ percentile, *SE* Standard error

The mean EQ VAS score for all was 81.4 (SE = 1.230) when people filled in the EQ-5D-5L and 82.2 (SE = 1.110) when people filled in the EQ-5D-3L (Z = 74.61, *p* < 0.0001). The mean time between the two administrations of EQ VAS was 43.6 min (SD = 104.01). 31.9% of the values people marked on the visual analogue scale differed between the two times the EQ VAS was administered. 9.3% of the values had a difference of ± 5 points and 3.4% of the values had a difference of ± 10 points. Differences higher than 10 points were recorded in only 5% of the answers that differed. Respondents older than 45 were responsible for 81.8% of the ± 10 points differences and for 75.9% of the ± 5 points differences between the two administrations of the EQ VAS.

We estimated population norms for the EQ VAS based on the values people reported when filling in the EQ-5D-5L questionnaire. In Table 3 we present the distribution of EQ VAS scores by age groups and sex. The EQ VAS scores decreased with age from age 35 onwards for all and when separated by sex. Men consistently reported better health than women across all age groups with one exception: age group 25–34. For this age group men reported worse health status than women with a difference as high as 7.3 points.

**Table 3:** Population norms for the EQ VAS for all, men and women, by age groups (weighted sample)

Age group	Percentiles	All	Men	Women
**18–24**	**Mean (SE)**	90.3 (1.311)	90.6 (1.653)	89.8 (1.859)
	**95%CI**	87.7—92.8	87.4—93.8	86.2—93.5
	**P 25**	85	85	80
	**Median**	93	90	95
	**P 75**	98	98	99
**25–34**	**Mean (SE)**	88.2 (1.990)	84.4 (15.63)	91.7 (1.083)
	**95%CI**	84.3—92.1	85.1—90.9	89.5—93.8
	**P 25**	85	80	90
	**Median**	91	90	95
	**P 75**	95	95	97
**35–44**	**Mean (SE)**	88.3 (1.220)	88.9 (1.316)	87.7 (1.583)
	**95%CI**	86.0—90.7	86.3—91.5	84.6—90.8
	**P 25**	85	85	85
	**Median**	90	90	90
	**P 75**	95	95	95
**45–54**	**Mean (SE)**	84.1 (0.951)	84.9 (1.087)	83.4 (1.258)
	**95%CI**	82.3—86.0	82.8—87.0	80.9—85.8
	**P 25**	80	80	80
	**Median**	89	90	87
	**P 75**	90	92	90
**55–64**	**Mean (SE)**	77.6 (1.180)	79.5 (1.438)	76.0 (1.716)
	**95%CI**	75.3—80.0	76.7—82.3	72.7—79.4
	**P 25**	70	75	70
	**Median**	80	80	80
	**P 75**	90	90	90
**65–74**	**Mean (SE)**	68.2 (2.231)	71.3 (2.312)	66.0 (2.753)
	**95%CI**	63.9—72.6	66.8—75.8	60.6—71.4
	**P 25**	60	61	70
	**Median**	70	70	70
	**P 75**	80	80	80
**75 or more**	**Mean (SE)**	60.4 (3.079)	62.4 (3.908)	59.3 (3.316)
	**95%CI**	54.4—66.5	54.7—70.0	52.8—65.8
	**P 25**	50	50	50
	**Median**	60	63	60
	**P 75**	77	75	80

*CI* Confidence interval, *P 25* 25^th^ percentile, *P 75* 75^th^ percentile, *SE* Standard error

**Fig. 2:**
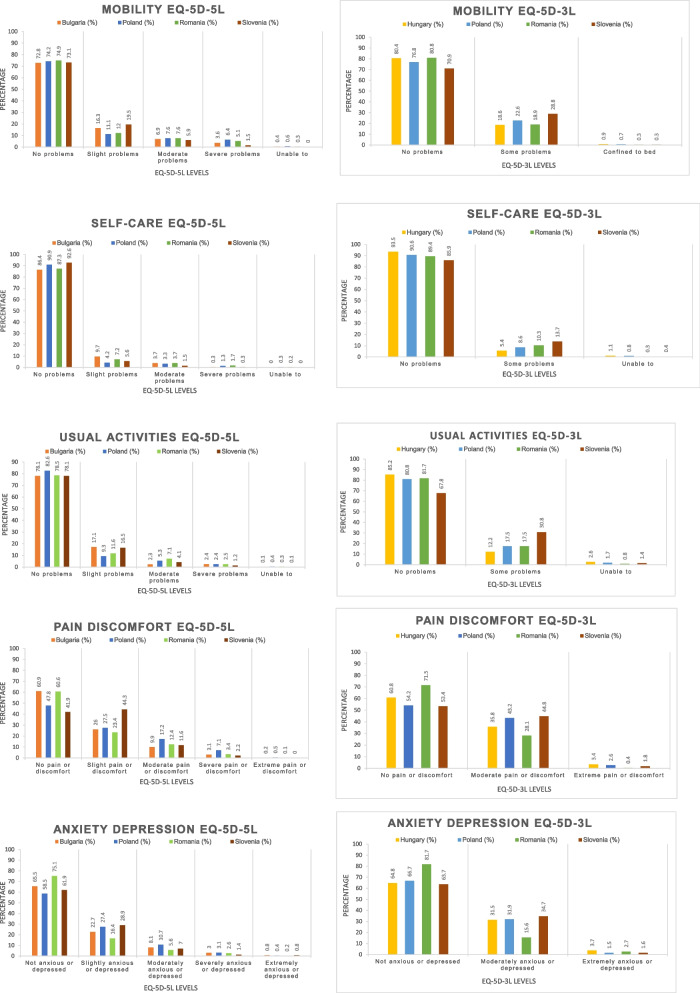
Percentage of people reporting problems in the EQ-5D-5L and EQ-5D-3L dimensions by country. Source: Encheva et al. 2020 [27]; Golicki et al. 2017 [28]; Prevolnik Rupel et al. 2020 [29]; Szende et al. 2003 [30]; Golicki et al. 2015 [31]; Prevolnik Rupel et al. 2020 [32]

As seen in Fig. 2, across all countries compared, the highest percentage of people with problems was registered for the pain/discomfort dimension for both questionnaires and the lowest for the self-care dimension. Romania and Bulgaria had a very similar distribution of people reporting no problems across all dimensions of EQ-5D-5L with the exception of the anxiety/depression dimension. All compared countries had similar percentages of people reporting no problems for the mobility, self-care and usual activities dimensions of the EQ-5D-5L. Romania stood out as the country with the highest and second highest percentage of people reporting no problems with anxiety/depression or pain/discomfort dimensions for both questionnaires.

## Discussion

Our study is the first one to have determined the HRQoL of the general population in Romania using an internationally validated HRQoL questionnaire, the EQ-5D. Furthermore, we have estimated, for the first time, population norms for the Romanian versions of EQ-5D-5L, EQ-5D-3L, their corresponding indexes, and the EQ VAS. These can now be used by health care professionals, decision makers, and researchers to better capture and reflect the health status of the people living in Romania.

We found differences between the values people reported on the visual analogue scale of the EQ VAS at the beginning and the end of our survey. This is in line with other study’s results that has shown that in older clinical populations changes in people’s self-report of HRQoL can occur even if there is no substantial change in their underlying health status [33]. In our case, the majority of differences between the two administrations of the EQ VAS occurred in older respondents as well. Changes in people’s self-reported health between the two times respondents filled in the EQ VAS might be due to the fact that in between the two administrations they were exposed to different health states, including the worse health state 55555, that they had to value as part of the composite time trade-off (cTTO) and discrete choice experiment (DCE) tasks. This is called context bias and has been documented by others in other studies that used the EQ VAS as well [34–36]. Hence, we decided to estimate population norms for the EQ VAS based on people’s answers when filling in the EQ-5D-5L at the beginning of our survey.

The anxiety/depression dimension was the only one for which young people reported a high percentage of problems when compared with the other dimensions of the EQ-5D-5L or the EQ-5D-3L for which problems generally increased with age. Nevertheless, this is in line with reports from other Eastern European countries, such as Slovenia [29], where the same phenomenon was observed or Moscow [37], where a similar pattern was found in young women. In contrast, in Poland this pattern was not observed, and problems with anxiety/depression increased with age [28]. Further research is needed to understand why common mental health disorders are on the rise among young people in Romania.

Older men in Romania (> 65 years old) tended to report fewer health problems in almost all dimensions of both questionnaires. This is in agreement with the general consensus in literature that women tend to report worse health than men [38]. However, for the self-care dimension men reported more problems than women for age groups 25–34, 45–54 and 55–64 for the EQ-5D-5L and for all age groups with the exception of the age group 55–64 for the EQ-5D-3L. This finding for the self-care domain of the EQ-5D-5L is similar with results obtained in one overlapping age group in other countries such as Norway [39] or Ireland [40].

We compared our EQ-5D data with different EQ-5D population surveys from different CEE countries. These population surveys were conducted over a period of almost ten years and used a variety of modes of administration from paper to web based and face-to-face administrations. Even though the mode of administration might not have impacted our results given the simplicity of EQ-5D questions [25] and the equivalence between paper, screen-based, and phone-based formats of the EQ-5D-5L [41, 42], HRQoL in general and EQ-5D ratings and values could have changed over time in the selected countries. These aspects should be taken into account when interpreting our results, even though we believe that differences observed between countries might not be fully explained only by methodological differences between surveys.

### Strengths and limitations

Several strengths of our survey need to be acknowledged. Overall, we had a very low rate of missing data in spite of the relatively long duration of the interview. We recruited and included respondents from all regions of Romania, leading to a final good sample size that was larger than the one used to estimate, for example, the population norms for SF-36 [18]. Finally, our survey was computer-assisted enabling the collection of more accurate and better-quality data [43].

However, a number of caveats need to be noted regarding our survey. First of all, our sample included more women (65%) and more people from urban areas (73.5%) than national average statistics (52% women; 55.2% urban population according to the 2011 census). One explanation behind the lower numbers of men recruited in our sample might be migration. Migration has been ongoing in Romania since the fall of Communism in 1990 [44] and, in recent years, it has been steadily increasing with exact numbers being hard to estimate due to freedom of movement within the EU [45]. Nevertheless, a 2018 report from the World Bank estimated that approximately 15.4% to 25.6% of the country’s total population lived and worked abroad [46] and some 2019 national statistics estimated that the majority of migrants (54.3%) were men, 83% of them having ages between 18–44 years [47]. Also, the time the interviews were performed might explain why more women were included in our sample than men. 66% of the interviews were performed during working hours (8am to 4 pm), a time when women were more likely to be at home than men given that they are less often employed or work as housewives. 2019 national statistics [48] show that employment rates are higher in men than in women in Romania (74.6% vs 56.8%) and that more men than women work longer hours than the 40 h legal length of the working week (13.3% vs 8.2%). Respondent selection within household might be another reason why men are underrepresented in our sample, as interviewers could have potentially been more prone to select the first person who agreed to participate rather than the person who would have his or hers birthday next [49]. Nevertheless, in the face-to-face trainings, interviewers were explained how to perform the next birthday selection and were reminded of the importance of this type of selection during feedback calls. Even though our raw sample presented these imbalances in terms of residence area and sex, we managed to correct these by using survey weights. Finally, in spite of the use of survey weights, we still expect some differences to exist between our sample and the general population of Romania, given that official data, such as the 2011 census or more recent reports issued by the National Institute of Statistics, do not fully take into consideration the emigration phenomenon [49]. More recent data on migration will become available at the end of 2023 when the data of the 2021 census has been fully processed [50].

By design, our survey had a pre-set, non-randomized order in which the five-block sections of the interview were displayed on the screen. Given that the EQ-5D-3L was completed towards the end of the interview, after EQ-5D-5L and all valuation tasks, we cannot exclude the fact that respondents’ answers might have been influenced by the previous sections of the survey. Additionally, the extended length of the interview might have made respondents more prone to fatigue and satisficing towards the end of the survey affecting the quality of their answers, especially for the EQ-5D-3L. However, both versions of the EQ-5D are simple to use and considered to be cognitively undemanding [51]. Moreover, a recent national health survey conducted in Catalonia showed that administering both versions of the questionnaire in the same survey does not seem to affect responses to the questionnaire placed second (in the case of the respective study, the EQ-5D-5L) [52].

## Conclusion

In this study we developed for the first time population norms for the Romanian versions of the EQ-5D-5L, EQ-5D-3L, their indices and the EQ VAS. This was done as part of a wider study that aimed to develop value sets for both the EQ-5D-5L and the EQ-5D-3L in Romania. The results of our study should further encourage the use of the EQ-5D in healthcare and research settings in Romania and will provide a valuable resource for those interested in comparing self-reported health across different populations in Romania or across different countries in the region.

## Supplementary Information

**Additional file 1: Supplementary Table 1.** Romanian population norms for the EQ-5D-5L dimensions by age groups (weighted sample). **Supplementary Table 2.** Romanian population norms for the EQ-5D-3L dimensions by age groups (weighted sample). **Supplementary Table 3.** Romanian population norms for men for the EQ-5D-5L dimensions by age groups (weighted sample). **Supplementary Table 4.** Romanian population norms for men for the EQ-5D-3L dimensions by age groups (weighted sample). **Supplementary Table 5.** Romanian population norms for women for the EQ-5D-5L dimensions by age groups (weighted sample). **Supplementary Table 6.** Romanian population norms for women for the EQ-5D-3L dimensions by age groups (weighted sample). **Supplementary Table 7.** Percentage of people reporting problems in the EQ-5D-5L and EQ-5D-3L dimensions by place of residence (weighted sample). **Supplementary Table 8.** Most frequently reported EQ-5D-5L health states and their mean index values and EQ VAS scores. Weighted sample. **Supplementary Table 9.** Most frequently reported EQ-5D-3L health states and their mean index values and EQ VAS scores. Weighted sample.

## Data Availability

The data used for this manuscript are available from the corresponding author upon reasonable request.

## Abbreviations

cTTO: Composite time trade-off
DCE: Discrete choice experiment
HRQoL: Health-related quality of life
PROMS: Patient-reported outcome measures
VAS: Visual analogue scale
