# Supplementary material for: Population norms for the EQ-5D-3L and EQ-5D-5L in Romania
Source: Health Qual Life Outcomes. 2023 Jul 29;21:80. doi: 10.1186/s12955-023-02144-8 (PMC10386277; doi:10.1186/s12955-023-02144-8)
Supplement: Supplementary file 1 — Additional file 1: Supplementary Table 1. Romanian population norms for the EQ-5D-5L dimensions by age groups (weighted sample). Supplementary Table 2. Romanian population norms for the EQ-5D-3L dimensions by age groups (weighted sample). Supplementary Table 3. Romanian population norms for men for the EQ-5D-5L dimensions by age groups (weighted sample). Supplementary Table 4. Romanian population norms for men for the EQ-5D-3L dimensions by age groups (weighted sample). Supplementary Table 5. Romanian population norms for women for the EQ-5D-5L dimensions by age groups (weighted sample). Supplementary Table 6. Romanian population norms for women for the EQ-5D-3L dimensions by age groups (weighted sample). Supplementary Table 7. Percentage of people reporting problems in the EQ-5D-5L and EQ-5D-3L dimensions by place of residence (weighted sample). Supplementary Table 8. Most frequently reported EQ-5D-5L health states and their mean index values and EQ VAS scores. Weighted sample. Supplementary Table 9. Most frequently reported EQ-5D-3L health states and their mean index values and EQ VAS scores. Weighted sample. [file 12955_2023_2144_MOESM1_ESM.docx]

Population norms the EQ-5D-3L and EQ-5D-5L in Romania

Elena Olariu^1^, Raluca Caplescu^2^, Luke Vale^1^, Ileana Gabriela Niculescu-Aron^2^, Yemi Oluboyede^1*^, Marian Sorin Paveliu ^3,4**^

^1^ Health Economics Group, Population Health Sciences Institute, Newcastle University, Newcastle upon Tyne, United Kingdom

^2^ Department of Statistics and Econometrics, Bucharest University of Economic Studies, Bucharest, 010374, Romania

^3^ Department of Pharmacology, Titu Maiorescu University, Bucharest, 031593, Romania

^4^ Romanian Academic Society, Bucharest, 020071, Romania

* Corresponding author for EQ-5D-5L population norms

Address for correspondence: Elena Olariu, PhD, Population Health Sciences Institute, Baddiley-Clark Building, Richardson Road, Newcastle upon Tyne, NE2 4AX, United Kingdom. Email: elena.olariu@ncl.ac.uk. Telephone: +44 (0)191 208 6949.

** Corresponding author for EQ-5D-3L population norms

Address for correspondence: Sorin Paveliu MD, PhD, Faculty of General Medicine, Titu Maiorescu University, 67A Gheorghe Petrascu Str., district 3, Bucharest, code 031593, Romania. Email: marian.paveliu@prof.utm.ro. Telephone: 0040726.200.627

Funding: The estimation of EQ-5D-5L population norms received funding from the European Union’s Horizon 2020 research and innovation programme under the Marie Skłodowska-Curie grant agreement No 748612. The estimation of EQ-5D-3L population norms received funding as part of a project run through the Romanian Operational Programme “Administrative Capacity” (POCA 2014-2020)

Keywords: EQ-5D-3L; EQ-5D-5L; Romania; Health-Related Quality of Life; Population Norms

Annex

**Supplementary table 1 - Romanian population norms for the EQ-5D-5L dimensions by age groups (weighted sample)**

| **Dimensions** | | **18-24** | | **25-34** | | **35-44** | | **45-54** | | **55-64** | | **65-74** | | **75 or more** | | **Total** | | |
| --- | --- | --- | --- | --- | --- | --- | --- | --- | --- | --- | --- | --- | --- | --- | --- | --- | --- | --- |
|  |  | **n=177** | | **n=293** | | **n=333** | | **n=244** | | **n=273** | | **n=182** | | **n=148** | | **n=1649** | | |
|  |  | **%** | **SE** | **%** | **SE** | **%** | **SE** | **%** | **SE** | **%** | **SE** | **%** | **SE** | **%** | **SE** | **%** | **SE** |  |
| Mobility | Level 1 | 99.5 | 0.427 | 92.7 | 2.921 | 91.2 | 3.446 | 82.3 | 4.263 | 61.3 | 4.252 | 45 | 4.667 | 23.1 | 5.242 | 74.9 | 2.683 |  |
|  | Level 2 | 0 |  | 4.1 | 1.887 | 6.1 | 2.437 | 12 | 2.981 | 22.4 | 3.857 | 24 | 4.075 | 21.6 | 4.905 | 12 | 1.531 |  |
|  | Level 3 | 0 |  | 0.7 | 0.672 | 1.2 | 0.929 | 4.5 | 1.494 | 10.6 | 2.214 | 20.1 | 3.094 | 29.4 | 5.972 | 7.6 | 1.29 |  |
|  | Level 4 | 0.5 | 0.427 | 2.5 | 2.282 | 1.5 | 1.130 | 1.2 | 0.664 | 5.4 | 1.536 | 9.8 | 2.941 | 24 | 4.869 | 5.1 | 1.032 |  |
|  | Level 5 | 0 |  | 0 |  | 0 |  | 0 |  | 0.3 | 0.278 | 1.1 | 0.644 | 1.9 | 1.644 | 0.3 | 0.145 |  |
| Self-Care | Level 1 | 99.5 | 0.427 | 96.5 | 2.377 | 96.2 | 2.314 | 93 | 2.879 | 84.8 | 2.578 | 74.3 | 3.561 | 45.3 | 5.554 | 87.3 | 1.505 |  |
|  | Level 2 | 0 |  | 0 |  | 2.7 | 1.834 | 4.7 | 1.675 | 11.4 | 2.143 | 16 | 2.508 | 25.1 | 5.031 | 7.2 | 0.804 |  |
|  | Level 3 | 0.5 | 0.427 | 1.3 | 0.816 | 0.5 | 0.508 | 2.3 | 1.361 | 2.6 | 1.070 | 8.2 | 1.892 | 18 | 7.098 | 3.7 | 0.852 |  |
|  | Level 4 | 0 |  | 2.2 | 2.244 | 0.5 | 0.462 | 0 |  | 1.2 | 0.687 | 0.9 | 0.709 | 9.7 | 3.348 | 1.7 | 0.641 |  |
|  | Level 5 | 0 |  | 0 |  | 0 |  | 0 |  | 0 |  | 0.5 | 0.489 | 1.9 | 1.644 | 0.2 | 0.125 |  |
| Usual Activities | Level 1 | 98.4 | 0.741 | 95.4 | 2.450 | 91.4 | 2.894 | 86.6 | 2.673 | 69.1 | 3.954 | 52.3 | 5.125 | 27.8 | 4.570 | 78.5 | 1.994 |  |
|  | Level 2 | 1.1 | 0.707 | 1.6 | 1.014 | 6.6 | 1.850 | 8.8 | 1.677 | 22.6 | 4.203 | 27.2 | 4.184 | 20.8 | 5.832 | 11.6 | 1.192 |  |
|  | Level 3 | 0.5 | 0.427 | 0 |  | 2 | 1.435 | 4.4 | 1.515 | 7.2 | 1.897 | 14.8 | 2.728 | 36 | 5.897 | 7.1 | 1.262 |  |
|  | Level 4 | 0 |  | 2.9 | 2.318 | 0 |  | 0.300 | 0.326 | 1.2 | 0.836 | 4.8 | 2.471 | 13.5 | 3.046 | 2.5 | 0.636 |  |
|  | Level 5 | 0 |  | 0 |  | 0 |  | 0 |  | 0 |  | 0.9 | 0.584 | 1.9 | 1.655 | 0.3 | 0.13 |  |
| Pain/ Discomfort * | Level 1 | 82.9 | 5.115 | 81.3 | 4.325 | 80.9 | 4.396 | 62.4 | 3.452 | 40.2 | 3.828 | 28.7 | 4.283 | 21.4 | 3.362 | 60.6 | 2.58 |  |
|  | Level 2 | 14.8 | 5.249 | 11.9 | 3.774 | 15.6 | 2.962 | 27.5 | 2.600 | 40.3 | 3.516 | 31.7 | 4.117 | 26.2 | 4.576 | 23.4 | 1.443 |  |
|  | Level 3 | 0.6 | 0.526 | 4.3 | 1.531 | 3 | 1.562 | 8.8 | 2.150 | 16.5 | 2.340 | 32.2 | 4.326 | 38.3 | 3.425 | 12.4 | 1.595 |  |
|  | Level 4 | 1.7 | 1.341 | 2.6 | 2.290 | 0.5 | 0.462 | 1.3 | 0.779 | 3 | 1.255 | 6.8 | 2.483 | 13.5 | 3.924 | 3.4 | 0.83 |  |
|  | Level 5 | 0 |  | 0 |  | 0 |  | 0 |  | 0 |  | 0.5 | 0.481 | 0.6 | 0.627 | 0.1 | 0.079 |  |
| Anxiety/ Depression ** | Level 1 | 73.9 | 5.899 | 80.9 | 4.362 | 85.8 | 2.730 | 77.5 | 4.006 | 73.2 | 4.591 | 66.9 | 3.687 | 51.1 | 7.447 | 75.1 | 2.454 |  |
|  | Level 2 | 22.2 | 5.700 | 11 | 2.458 | 10.1 | 1.393 | 16.5 | 3.065 | 18.8 | 4.227 | 18.6 | 3.478 | 27.3 | 4.316 | 16.4 | 1.637 |  |
|  | Level 3 | 3.9 | 1.585 | 3 | 1.813 | 3.1 | 1.936 | 6 | 2.475 | 7 | 1.882 | 9.3 | 2.335 | 10.5 | 3.397 | 5.6 | 0.94 |  |
|  | Level 4 | 0 |  | 5.2 | 2.786 | 1 | 0.608 | 0 |  | 0.4 | 0.405 | 3.8 | 1.719 | 11 | 2.691 | 2.6 | 0.718 |  |
|  | Level 5 | 0 |  | 0 |  | 0 |  | 0 |  | 0.5 | 0.586 | 1.4 | 0.990 | 0 |  | 0.2 | 0.155 |  |

*SE*, standard error;

Level 1 (no problems), Level 2 (slight problems), Level 3 (moderate problems), Level 4 (severe problems), Level 5 (unable to)

* Level 1 (no pain or discomfort), Level 2 (slight pain or discomfort), Level 3 (moderate pain or discomfort), Level 4 (severe pain or discomfort), Level 5 (extreme pain or discomfort)

** Level 1 (not anxious or depressed), Level 2 (slightly anxious or depressed), Level 3 (moderately anxious or depressed), Level 4 (severely anxious or depressed), Level 5 (extremely anxious or depressed)

**Supplementary table 2 - Romanian population norms for the EQ-5D-3L dimensions by age groups (weighted sample)**

| **Dimensions** | | **18-24** | | **25-34** | | **35-44** | | **45-54** | | **55-64** | | **65-74** | | **75 or more** | | **Total** | |
| --- | --- | --- | --- | --- | --- | --- | --- | --- | --- | --- | --- | --- | --- | --- | --- | --- | --- |
|  |  | **n=177** | | **n=293** | | **n=333** | | **n=244** | | **n=273** | | **n=182** | | **n=148** | | **n=1649** | |
|  |  | **%** | **SE** | **%** | **SE** | **%** | **SE** | **%** | **SE** | **%** | **SE** | **%** | **SE** | **%** | **SE** | **%** | **SE** |
| Mobility* | Level 1 | 98.9 | 0.635 | 96.8 | 2.352 | 94.9 | 2.359 | 89.2 | 2.550 | 73.5 | 3.587 | 54.3 | 4.388 | 28.1 | 4.438 | 80.8 | 2.101 |
|  | Level 2 | 1.1 | 0.635 | 3.2 | 2.352 | 5.1 | 2.359 | 10.8 | 2.550 | 26.5 | 3.587 | 45.2 | 4.183 | 69.4 | 4.721 | 18.9 | 2.073 |
|  | Level 3 | 0 |  | 0 |  | 0 |  | 0 |  | 0 |  | 0.5 | 0.489 | 2.5 | 1.809 | 0.3 | 0.138 |
| Self-Care | Level 1 | 99.5 | 0.427 | 96.5 | 2.377 | 97.9 | 1.823 | 95 | 1.847 | 88.5 | 3.086 | 80.5 | 3.928 | 47.4 | 5.889 | 89.4 | 1.288 |
|  | Level 2 | 0.5 | 0.427 | 3.5 | 2.377 | 2.1 | 1.823 | 5 | 1.847 | 11.5 | 3.086 | 19 | 3.599 | 50.1 | 6.092 | 10.3 | 1.287 |
|  | Level 3 | 0 |  | 0 |  | 0 |  | 0 |  | 0 |  | 0.5 | 0.489 | 2.5 | 1.809 | 0.3 | 0.138 |
| Usual Activities | Level 1 | 98.4 | 0.741 | 96.5 | 2.377 | 95.1 | 2.162 | 88.2 | 2.792 | 76.2 | 2.967 | 62 | 4.252 | 26.4 | 4.316 | 81.7 | 1.686 |
|  | Level 2 | 1.6 | 0.741 | 3.5 | 2.377 | 4.9 | 2.162 | 11.8 | 2.792 | 23.2 | 2.893 | 36.2 | 3.770 | 67.8 | 5.536 | 17.4 | 1.671 |
|  | Level 3 | 0 |  | 0 |  | 0 |  | 0 |  | 0.6 | 0.633 | 1.8 | 0.810 | 5.7 | 2.394 | 0.8 | 0.203 |
| Pain/ Discomfort ** | Level 1 | 95.2 | 1.641 | 90.3 | 2.835 | 87.5 | 3.256 | 76.6 | 3.562 | 54.6 | 4.198 | 39.1 | 4.652 | 32.2 | 4.147 | 71.5 | 2.341 |
|  | Level 2 | 4.8 | 1.641 | 9.7 | 2.835 | 12.5 | 3.256 | 23.4 | 3.562 | 45.4 | 4.198 | 60 | 4.351 | 64.6 | 4.091 | 28.1 | 2.255 |
|  | Level 3 | 0 |  | 0 |  | 0 |  | 0 |  | 0 |  | 0.9 | 0.561 | 3.2 | 1.908 | 0.4 | 0.209 |
| Anxiety/ Depression *** | Level 1 | 87.9 | 2.572 | 87.8 | 3.379 | 89.9 | 3.034 | 82.3 | 3.360 | 77.8 | 4.427 | 73.9 | 5.733 | 59.7 | 6.638 | 81.7 | 2.403 |
|  | Level 2 | 9.7 | 2.161 | 11.6 | 3.133 | 6.9 | 2.092 | 15.9 | 3.079 | 18.9 | 4.509 | 21.1 | 4.372 | 36.6 | 6.463 | 15.6 | 1.851 |
|  | Level 3 | 2.4 | 1.668 | 0.6 | 1.668 | 3.3 | 2.738 | 1.8 | 1.779 | 3.3 | 1.877 | 5 | 2.799 | 3.8 | 3.036 | 2.7 | 1.719 |

*SE*, standard error

Level 1 (no problems), Level 2 (some problems), Level 3 (unable to)

* Level 1 (no problems), Level 2 (some problems), Level 3 (confined to bed)

** Level 1 (no pain or discomfort), Level 2 (moderate pain or discomfort), Level 3 (extreme pain or discomfort)

*** Level 1 (not anxious or depressed), Level 2 (moderately anxious or depressed), Level 3 (extremely anxious or depressed)

**Supplementary table 3 - Romanian population norms for men for the EQ-5D-5L dimensions by age groups (weighted sample)**

| **Dimensions** | | **18-24** | | **25-34** | | **35-44** | | **45-54** | | **55-64** | | **65-74** | | **75 or more** | | **Total** | |
| --- | --- | --- | --- | --- | --- | --- | --- | --- | --- | --- | --- | --- | --- | --- | --- | --- | --- |
|  |  | **n=101** | | **n=139** | | **n=169** | | **n=122** | | **n=128** | | **n=77** | | **n=55** | | **n=792** | |
|  |  | **%** | **SE** | **%** | **SE** | **%** | **SE** | **%** | **SE** | **%** | **SE** | **%** | **SE** | **%** | **SE** | **%** | **SE** |
| Mobility | Level 1 | 100 | 0.000 | 88.3 | 5.353 | 93.5 | 3.340 | 82.7 | 4.316 | 62.3 | 5.450 | 50.5 | 5.091 | 21 | 6.965 | 77.5 | 2.927 |
|  | Level 2 |  |  | 7 | 3.410 | 2.6 | 1.956 | 10.6 | 3.039 | 21.1 | 4.520 | 27.2 | 5.522 | 23.2 | 7.637 | 11.1 | 1.647 |
|  | Level 3 |  |  |  |  | 1.9 | 1.803 | 5.1 | 2.493 | 12.1 | 2.959 | 12 | 5.604 | 38.3 | 9.397 | 7.0 | 1.470 |
|  | Level 4 |  |  | 4.7 | 4.749 | 1.9 | 2.095 | 1.6 | 1.138 | 4.5 | 2.246 | 10.3 | 4.028 | 17.5 | 5.028 | 4.4 | 1.189 |
|  | Level 5 |  |  |  |  |  |  |  |  |  |  |  |  |  |  |  |  |
| Self-Care | Level 1 | 100 | 0.000 | 95.3 | 4.750 | 98.1 | 1.803 | 91.1 | 3.285 | 81.6 | 4.637 | 77 | 5.506 | 49.2 | 7.261 | 88.6 | 1.930 |
|  | Level 2 |  |  |  |  | 1.9 | 1.803 | 5.4 | 1.991 | 12.1 | 3.505 | 13 | 4.537 | 19.5 | 7.081 | 5.8 | 1.040 |
|  | Level 3 |  |  |  |  |  |  | 3.5 | 2.165 | 4.9 | 2.064 | 9 | 3.702 | 17.5 | 6.758 | 3.4 | 0.902 |
|  | Level 4 |  |  | 4.7 | 4.749 |  |  |  |  | 1.3 | 1.430 | 1 | 1.055 | 13.8 | 6.473 | 2.1 | 1.020 |
|  | Level 5 |  |  |  |  |  |  |  |  |  |  |  |  |  |  |  |  |
| Usual Activities | Level 1 | 98.9 | 0.954 | 95.3 | 4.749 | 93.5 | 3.090 | 85.6 | 3.527 | 69.1 | 6.527 | 61.6 | 7.615 | 42 | 7.255 | 82.7 | 1.975 |
|  | Level 2 | 1.1 | 0.954 |  |  | 4.5 | 2.567 | 8.3 | 2.819 | 26 | 6.409 | 22.7 | 7.144 | 16 | 7.771 | 9.9 | 1.983 |
|  | Level 3 |  |  |  |  | 1.9 | 1.803 | 5.4 | 2.851 | 3.6 | 1.998 | 9.3 | 5.260 | 29.8 | 9.118 | 4.8 | 1.515 |
|  | Level 4 |  |  | 4.7 | 4.749 |  |  | 0.6 | 0.672 | 1.3 | 1.300 | 6.4 | 3.799 | 12.2 | 4.941 | 2.6 | 1.033 |
|  | Level 5 |  |  |  |  |  |  |  |  |  |  |  |  |  |  |  |  |
| Pain/ | Level 1 | 77.4 | 9.394 | 78.9 | 6.985 | 84.6 | 3.757 | 62.9 | 4.392 | 51 | 5.800 | 34.1 | 7.499 | 28 | 6.148 | 65.1 | 2.892 |
| Discomfort * | Level 2 | 21.5 | 9.568 | 14 | 6.578 | 12.8 | 3.519 | 27.2 | 4.650 | 34.2 | 4.763 | 38 | 8.226 | 36.9 | 8.058 | 23.9 | 2.664 |
|  | Level 3 | 1.1 | 0.914 | 1.6 | 1.160 | 2.6 | 1.881 | 8.3 | 3.198 | 11.2 | 3.594 | 27.9 | 6.054 | 28 | 6.119 | 8.7 | 1.299 |
|  | Level 4 |  |  | 5.4 | 4.854 |  |  | 1.6 | 1.190 | 3.6 | 2.300 |  |  | 7 | 3.560 | 2.3 | 0.924 |
|  | Level 5 |  |  |  |  |  |  |  |  |  |  |  |  |  |  |  |  |
| Anxiety/ Depression ** | Level 1 | 65.6 | 10.930 | 79.7 | 7.190 | 91.3 | 2.663 | 79.2 | 4.247 | 78.4 | 5.754 | 74 | 4.800 | 51 | 11.460 | 77.5 | 2.853 |
|  | Level 2 | 32.3 | 10.335 | 11 | 3.365 | 4.9 | 1.582 | 13.2 | 2.588 | 16.2 | 5.454 | 17.6 | 5.485 | 35.1 | 8.372 | 15.9 | 2.059 |
|  | Level 3 | 2.2 | 1.050 |  |  | 3.8 | 2.467 | 7.6 | 3.180 | 4.5 | 1.912 | 8.3 | 3.978 | 5.2 | 3.578 | 4.2 | 1.119 |
|  | Level 4 |  |  | 9.3 | 5.361 |  |  |  |  | 0.9 | 0.910 |  |  | 8.7 | 3.935 | 2.4 | 1.108 |
|  | Level 5 |  |  |  |  |  |  |  |  |  |  |  |  |  |  |  |  |

*SE*, standard error;

Level 1 (no problems), Level 2 (slight problems), Level 3 (moderate problems), Level 4 (severe problems), Level 5 (unable to)

* Level 1 (no pain or discomfort), Level 2 (slight pain or discomfort), Level 3 (moderate pain or discomfort), Level 4 (severe pain or discomfort), Level 5 (extreme pain or discomfort)

** Level 1 (not anxious or depressed), Level 2 (slightly anxious or depressed), Level 3 (moderately anxious or depressed), Level 4 (severely anxious or depressed), Level 5 (extremely anxious or depressed)

**Supplementary table 4 - Romanian population norms for men for the EQ-5D-3L dimensions by age groups (weighted sample)**

| Dimensions | | **18-24** | | **25-34** | | **35-44** | | **45-54** | | **55-64** | | **65-74** | | **75 or more** | | **Total** | |
| --- | --- | --- | --- | --- | --- | --- | --- | --- | --- | --- | --- | --- | --- | --- | --- | --- | --- |
|  |  | **n=101** | | **n=139** | | **n=169** | | **n=122** | | **n=128** | | **n=77** | | **n=55** | | **n=792** | |
|  |  | **%** | **SE** | **%** | **SE** | **%** | **SE** | **%** | **SE** | **%** | **SE** | **%** | **SE** | **%** | **SE** | **%** | **SE** |
| Mobility* | Level 1 | 98.9 | 0.954 | 94.6 | 4.854 | 94.8 | 2.976 | 87.8 | 2.828 | 72.2 | 5.612 | 63.7 | 4.589 | 24.5 | 5.970 | 82.6 | 2.647 |
|  | Level 2 | 1.1 | 0.954 | 5.4 | 4.854 | 5.2 | 2.976 | 12.2 | 2.828 | 27.8 | 5.612 | 36.3 | 4.589 | 75.5 | 5.970 | 17.4 | 2.647 |
|  | Level 3 |  |  |  |  |  |  |  |  |  |  |  |  |  |  |  |  |
| Self-Care | Level 1 | 100 |  | 95.3 | 4.749 | 98.1 | 1.803 | 93.6 | 2.475 | 82.5 | 4.862 | 83.4 | 3.785 | 42.3 | 5.662 | 89.3 | 1.721 |
|  | Level 2 |  |  | 4.7 | 4.749 | 1.9 | 1.803 | 6.4 | 2.475 | 17.5 | 4.862 | 16.6 | 3.785 | 57.7 | 5.662 | 10.7 | 1.721 |
|  | Level 3 |  |  |  |  |  |  |  |  |  |  |  |  |  |  |  |  |
| Usual Activities | Level 1 | 98.9 | 0.954 | 95.3 | 4.749 | 94.2 | 3.243 | 89.1 | 3.373 | 74.9 | 5.119 | 66 | 6.817 | 36.9 | 8.033 | 84.4 | 2.184 |
|  | Level 2 | 1.1 | 0.954 | 4.7 | 4.749 | 5.8 | 3.243 | 10.9 | 3.373 | 23.8 | 4.470 | 33 | 6.730 | 63.1 | 8.033 | 15.3 | 2.231 |
|  | Level 3 |  |  |  |  |  |  |  |  | 1.3 | 1.300 | 1 | 1.055 |  |  | 0.3 | 0.226 |
| Pain/ Discomfort ** | Level 1 | 98.9 | 0.954 | 86.7 | 5.169 | 88.8 | 3.492 | 77.9 | 4.365 | 59.1 | 5.869 | 51.2 | 5.396 | 37 | 8.336 | 76.0 | 2.507 |
|  | Level 2 | 1.1 | 0.954 | 13.3 | 5.169 | 11.2 | 3.492 | 22.1 | 4.365 | 40.9 | 5.869 | 48.8 | 5.396 | 63 | 8.336 | 24.0 | 2.507 |
|  | Level 3 |  |  |  |  |  |  |  |  |  |  |  |  |  |  |  |  |
| Anxiety/ Depression *** | Level 1 | 89.2 | 3.006 | 88.3 | 5.416 | 89.9 | 4.440 | 80.5 | 4.342 | 82.1 | 6.583 | 82.8 | 4.893 | 63.3 | 10.774 | 84.3 | 2.735 |
|  | Level 2 | 7.6 | 2.734 | 11.7 | 5.416 | 5.9 | 2.710 | 16.9 | 4.210 | 13.9 | 6.245 | 13.3 | 4.045 | 34.9 | 10.669 | 12.9 | 2.130 |
|  | Level 3 | 3.2 | 2.743 |  |  | 4.2 | 3.994 | 2.6 | 2.515 | 4.1 | 2.849 | 3.9 | 4.006 | 1.8 | 1.889 | 2.9 | 2.221 |

*SE*, standard error

Level 1 (no problems), Level 2 (some problems), Level 3 (unable to)

* Level 1 (no problems), Level 2 (some problems), Level 3 (confined to bed)

** Level 1 (no pain or discomfort), Level 2 (moderate pain or discomfort), Level 3 (extreme pain or discomfort)

*** Level 1 (not anxious or depressed), Level 2 (moderately anxious or depressed), Level 3 (extremely anxious or depressed)

**Supplementary table 5 - Romanian population norms for women for the EQ-5D-5L dimensions by age groups (weighted sample)**

| **Dimensions** | | **18-24** | | **25-34** | | **35-44** | | **45-54** | | **55-64** | | **65-74** | | **75 or more** | | **Total** | |
| --- | --- | --- | --- | --- | --- | --- | --- | --- | --- | --- | --- | --- | --- | --- | --- | --- | --- |
|  |  | **n=76** | | **n=153** | | **n=163** | | **n=123** | | **n=145** | | **n=104** | | **n=93** | | **n=857** | |
|  |  | **%** | **SE** | **%** | **SE** | **%** | **SE** | **%** | **SE** | **%** | **SE** | **%** | **SE** | **%** | **SE** | **%** | **SE** |
| Mobility | Level 1 | 98.8 | 1.086 | 96.7 | 2.464 | 88.7 | 4.632 | 81.9 | 4.818 | 60.5 | 4.664 | 41.0 | 6.383 | 24.4 | 5.458 | 72.5 | 2.910 |
|  | Level 2 |  |  | 1.4 | 1.201 | 9.7 | 3.979 | 13.4 | 3.796 | 23.6 | 4.187 | 21.6 | 4.280 | 20.6 | 4.861 | 12.9 | 1.946 |
|  | Level 3 |  |  | 1.4 | 1.201 | 0.5 | 0.528 | 3.9 | 1.353 | 9.2 | 3.102 | 26.1 | 5.328 | 24.1 | 6.456 | 8.2 | 1.525 |
|  | Level 4 | 1.2 | 1.086 | 1.2 | 0.641 | 1.0 | 0.943 | 0.8 | 0.686 | 6.2 | 2.521 | 9.5 | 3.644 | 27.8 | 6.728 | 5.7 | 1.184 |
|  | Level 5 |  |  |  |  |  |  |  |  | 0.5 | 0.532 | 1.8 | 1.069 | 3.0 | 3.04 | 0.6 | 0.272 |
| Self-Care | Level 1 | 98.8 | 1.086 | 97.5 | 1.454 | 94.4 | 2.996 | 94.9 | 2.887 | 87.6 | 2.418 | 72.2 | 3.722 | 43.0 | 6.395 | 86.0 | 1.635 |
|  | Level 2 |  |  |  |  | 3.6 | 2.092 | 4.0 | 2.239 | 10.8 | 2.349 | 18.3 | 3.170 | 28.5 | 5.624 | 8.4 | 1.096 |
|  | Level 3 | 1.2 | 1.086 | 2.5 | 1.454 | 1.0 | 0.956 | 1.1 | 0.758 | 0.5 | 0.532 | 7.6 | 2.226 | 18.2 | 9.018 | 3.9 | 0.958 |
|  | Level 4 |  |  |  |  | 1.0 | 0.943 |  |  | 1.0 | 1.04 | 0.9 | 0.894 | 7.2 | 3.306 | 1.3 | 0.552 |
|  | Level 5 |  |  |  |  |  |  |  |  |  |  | 0.9 | 0.853 | 3.0 | 2.531 | 0.4 | 0.236 |
| Usual Activities | Level 1 | 97.7 | 1.481 | 95.5 | 1.701 | 89.2 | 3.861 | 87.5 | 3.141 | 69.0 | 4.119 | 45.5 | 5.977 | 19.3 | 4.902 | 74.6 | 2.776 |
|  | Level 2 | 1.2 | 1.103 | 3.1 | 1.928 | 8.7 | 3.131 | 9.2 | 2.727 | 19.6 | 4.101 | 30.5 | 4.250 | 23.7 | 5.489 | 13.2 | 1.548 |
|  | Level 3 | 1.2 | 1.086 |  |  | 2.1 | 1.296 | 3.3 | 1.612 | 10.3 | 2.450 | 18.9 | 4.583 | 39.7 | 6.643 | 9.3 | 1.598 |
|  | Level 4 |  |  | 1.4 | 1.201 |  |  |  |  | 1.0 | 1.040 | 3.7 | 1.924 | 14.3 | 3.801 | 2.4 | 0.558 |
|  | Level 5 |  |  |  |  |  |  |  |  |  |  | 1.5 | 1.04 | 3.0 | 2.546 | 0.5 | 0.247 |
| Pain/ | Level 1 | 90.2 | 3.546 | 83.4 | 4.456 | 77.1 | 6.517 | 61.8 | 4.748 | 30.7 | 4.652 | 24.7 | 3.550 | 17.4 | 3.659 | 56.5 | 3.688 |
| Discomfort * | Level 2 | 5.9 | 2.074 | 9.9 | 3.124 | 18.6 | 4.644 | 27.7 | 3.679 | 45.6 | 4.342 | 27.1 | 4.681 | 19.9 | 4.426 | 23.0 | 2.365 |
|  | Level 3 |  |  | 6.7 | 2.514 | 3.3 | 1.688 | 9.4 | 3.099 | 21.1 | 4.725 | 35.4 | 5.194 | 44.4 | 4.280 | 15.8 | 2.216 |
|  | Level 4 | 4.0 | 2.970 |  |  | 1.0 | 0.943 | 1.1 | 0.807 | 2.5 | 1.646 | 11.9 | 3.803 | 17.3 | 6.792 | 4.4 | 0.979 |
|  | Level 5 |  |  |  |  |  |  |  |  |  |  | 0.9 | 0.785 | 0.9 | 1.016 | 0.2 | 0.151 |
| Anxiety/ Depression ** | Level 1 | 85.0 | 4.997 | 82.0 | 5.578 | 80.1 | 3.960 | 75.8 | 4.736 | 68.6 | 5.278 | 61.6 | 5.898 | 51.3 | 7.941 | 73.0 | 3.062 |
|  | Level 2 | 8.6 | 4.106 | 11.0 | 3.604 | 15.5 | 2.753 | 19.8 | 4.766 | 21.2 | 4.780 | 19.3 | 4.554 | 22.7 | 4.976 | 16.9 | 2.218 |
|  | Level 3 | 6.3 | 3.294 | 5.7 | 3.178 | 2.3 | 1.432 | 4.4 | 2.526 | 9.2 | 2.912 | 10.0 | 2.397 | 13.6 | 4.672 | 6.9 | 1.087 |
|  | Level 4 |  |  | 1.4 | 1.201 | 2.1 | 1.263 |  |  |  |  | 6.7 | 2.922 | 12.4 | 2.613 | 2.8 | 0.788 |
|  | Level 5 |  |  |  |  |  |  |  |  | 1.0 | 1.047 | 2.4 | 1.603 |  |  | 0.5 | 0.291 |

*SE*, standard error;

Level 1 (no problems), Level 2 (slight problems), Level 3 (moderate problems), Level 4 (severe problems), Level 5 (unable to)

* Level 1 (no pain or discomfort), Level 2 (slight pain or discomfort), Level 3 (moderate pain or discomfort), Level 4 (severe pain or discomfort), Level 5 (extreme pain or discomfort)

** Level 1 (not anxious or depressed), Level 2 (slightly anxious or depressed), Level 3 (moderately anxious or depressed), Level 4 (severely anxious or depressed), Level 5 (extremely anxious or depressed)

**Supplementary table 6 - Romanian population norms for women for the EQ-5D-3L dimensions by age groups (weighted sample)**

| **Dimensions** | | **18-24** | | **25-34** | | **35-44** | | **45-54** | | **55-64** | | **65-74** | | **75 or more** | | **Total** | | |
| --- | --- | --- | --- | --- | --- | --- | --- | --- | --- | --- | --- | --- | --- | --- | --- | --- | --- | --- |
|  |  | **n=76** | | **n=153** | | **n=163** | | **n=123** | | **n=145** | | **n=104** | | **n=93** | | **n=857** | | |
|  |  | **%** | **SE** | **%** | **SE** | **%** | **SE** | **%** | **SE** | **%** | **SE** | **%** | **SE** | **%** | **SE** | **%** | **SE** |  |
| Mobility* | Level 1 | 98.8 | 1.086 | 98.8 | 0.891 | 95.1 | 2.567 | 90.5 | 3.228 | 74.7 | 4.168 | 47.2 | 6.295 | 30.3 | 4.953 | 79.2 | 2.427 |  |
|  | Level 2 | 1.2 | 1.086 | 1.2 | 0.891 | 4.9 | 2.567 | 9.5 | 3.228 | 25.3 | 4.168 | 51.9 | 6.137 | 65.7 | 5.141 | 20.3 | 2.426 |  |
|  | Level 3 |  |  |  |  |  |  |  |  |  |  | 0.9 | 0.853 | 4.0 | 2.765 | 0.5 | 0.259 |  |
| Self-Care | Level 1 | 98.8 | 1.086 | 97.5 | 1.454 | 97.7 | 1.907 | 96.4 | 1.672 | 93.8 | 1.912 | 78.3 | 5.297 | 50.4 | 7.103 | 89.5 | 1.335 |  |
|  | Level 2 | 1.2 | 1.086 | 2.5 | 1.454 | 2.3 | 1.907 | 3.6 | 1.672 | 6.2 | 1.912 | 20.7 | 4.802 | 45.6 | 7.230 | 10 | 1.360 |  |
|  | Level 3 |  |  |  |  |  |  |  |  |  |  | 0.9 | 0.853 | 4.0 | 2.765 | 0.5 | 0.259 |  |
| Usual Activities | Level 1 | 97.7 | 1.481 | 97.5 | 1.454 | 95.9 | 1.581 | 87.3 | 3.005 | 77.4 | 3.383 | 59.0 | 5.449 | 20.2 | 4.224 | 79.3 | 2.202 |  |
|  | Level 2 | 2.3 | 1.481 | 2.5 | 1.454 | 4.1 | 1.581 | 12.7 | 3.005 | 22.6 | 3.383 | 38.5 | 4.901 | 70.7 | 5.535 | 19.4 | 1.956 |  |
|  | Level 3 |  |  |  |  |  |  |  |  |  |  | 2.4 | 1.195 | 9.1 | 3.341 | 1.3 | 0.437 |  |
| Pain/ Discomfort ** | Level 1 | 90.2 | 3.239 | 93.5 | 2.317 | 86.2 | 4.163 | 75.4 | 4.529 | 50.6 | 4.947 | 30.1 | 4.629 | 29.3 | 4.776 | 67.3 | 2.965 |  |
|  | Level 2 | 9.8 | 3.239 | 6.5 | 2.317 | 13.8 | 4.163 | 24.6 | 4.529 | 49.4 | 4.947 | 68.4 | 4.281 | 65.5 | 4.57 | 31.9 | 2.835 |  |
|  | Level 3 |  |  |  |  |  |  |  |  |  |  | 1.5 | 0.913 | 5.1 | 3.412 | 0.7 | 0.394 |  |
| Anxiety/ Depression *** | Level 1 | 86.2 | 4.620 | 87.3 | 4.260 | 89.8 | 2.914 | 84.1 | 3.582 | 74.1 | 4.447 | 67.3 | 7.500 | 57.5 | 6.614 | 79.3 | 2.740 |  |
|  | Level 2 | 12.6 | 4.499 | 11.6 | 3.655 | 7.9 | 2.467 | 14.9 | 3.334 | 23.4 | 4.314 | 26.9 | 5.678 | 37.5 | 6.527 | 18.1 | 2.118 |  |
|  | Level 3 | 1.2 | 1.322 | 1.2 | 1.170 | 2.3 | 1.772 | 1.0 | 1.022 | 2.5 | 1.571 | 5.8 | 3.239 | 4.9 | 3.943 | 2.6 | 1.399 |  |

*SE*, standard error

Level 1 (no problems), Level 2 (some problems), Level 3 (unable to)

* Level 1 (no problems), Level 2 (some problems), Level 3 (confined to bed)

** Level 1 (no pain or discomfort), Level 2 (moderate pain or discomfort), Level 3 (extreme pain or discomfort)

*** Level 1 (not anxious or depressed), Level 2 (moderately anxious or depressed), Level 3 (extremely anxious or depressed)

**Supplementary table 7 - Percentage of people reporting problems in the EQ-5D-5L and EQ-5D-3L dimensions by place of residence (weighted sample)**

| **EQ-5D-5L** | | | | | | | | **EQ-5D-3L** | | | | | | | |
| --- | --- | --- | --- | --- | --- | --- | --- | --- | --- | --- | --- | --- | --- | --- | --- |
| **Dimensions** | | **Urban** | | **Rural** | | **Total** | | **Dimensions** | | **Urban** | | **Rural** | | **Total** | |
|  |  | **n=895** | | **n=754** | | **n=1649** | |  |  | **n=895** | | **n=754** | | **n=1649** | |
|  |  | **%** | **SE** | **%** | **SE** | **%** | **SE** |  |  | **%** | **SE** | **%** | **SE** | **%** | **SE** |
| Mobility | No problems | 82.8 | 2.295 | 65.6 | 5.516 | 74.9 | 2.683 | Mobility | No problems | 87.2 | 1.63 | 73.3 | 4.542 | 80.8 | 2.101 |
|  | Slight problems | 10.8 | 1.727 | 13.5 | 2.747 | 12 | 1.531 |  |  |  |  |  |  |  |  |
|  | Moderate problems | 4.0 | 0.677 | 11.9 | 2.804 | 7.6 | 1.290 |  | Some problems | 12.6 | 1.68 | 26.4 | 4.432 | 18.9 | 2.073 |
|  | Severe problems | 2.3 | 0.575 | 8.4 | 2.232 | 5.1 | 1.032 |  | Confined to bed | 0.2 | 0.135 | 0.4 | 0.258 | 0.3 | 0.138 |
|  | Unable to | 0.2 | 0.113 | 0.5 | 0.291 | 0.3 | 0.145 |  |  |  |  |  |  |  |  |
| Self-Care | No problems | 94.0 | 1.161 | 79.3 | 3.206 | 87.3 | 1.505 | Self-Care | No problems | 94.5 | 1.145 | 83.4 | 2.694 | 89.4 | 1.288 |
|  | Slight problems | 3.8 | 0.935 | 11.1 | 1.432 | 7.2 | 0.804 |  |  |  |  |  |  |  |  |
|  | Moderate problems | 1.9 | 0.689 | 5.8 | 1.802 | 3.7 | 0.852 |  | Some problems | 5.3 | 1.148 | 16.3 | 2.668 | 10.3 | 1.287 |
|  | Severe problems | 0.2 | 0.185 | 3.4 | 1.422 | 1.7 | 0.641 |  | Unable | 0.2 | 0.135 | 0.4 | 0.258 | 0.3 | 0.138 |
|  | Unable to | 0.1 | 0.093 | 0.4 | 0.258 | 0.2 | 0.125 |  |  |  |  |  |  |  |  |
| Usual Activities | No problems | 87.5 | 1.802 | 67.7 | 4.103 | 78.5 | 1.994 | Usual Activities | No problems | 89.4 | 1.492 | 72.6 | 3.564 | 81.7 | 1.686 |
|  | Slight problems | 8.4 | 1.621 | 15.5 | 1.879 | 11.6 | 1.192 |  |  |  |  |  |  |  |  |
|  | Moderate problems | 3.3 | 0.806 | 11.7 | 2.751 | 7.1 | 1.262 |  | Some problems | 10.1 | 1.453 | 26.1 | 3.545 | 17.4 | 1.671 |
|  | Severe problems | 0.7 | 0.249 | 4.7 | 1.377 | 2.5 | 0.636 |  | Unable | 0.4 | 0.237 | 1.3 | 0.359 | 0.8 | 0.203 |
|  | Unable to | 0.2 | 0.11 | 0.4 | 0.258 | 0.3 | 0.13 |  |  |  |  |  |  |  |  |
| Pain/ Discomfort | No pain or discomfort | 69.4 | 2.852 | 50.2 | 4.752 | 60.6 | 2.580 | Pain/ Discomfort | No pain or discomfort | 78.8 | 1.841 | 62.8 | 4.976 | 71.5 | 2.341 |
|  | Slight pain or discomfort | 21.6 | 1.977 | 25.6 | 2.148 | 23.4 | 1.443 |  |  |  |  |  |  |  |  |
|  | Moderate pain or discomfort | 7.5 | 1.215 | 18.3 | 3.361 | 12.4 | 1.595 |  | Moderate pain or discomfort | 21.0 | 1.77 | 36.6 | 4.802 | 28.1 | 2.255 |
|  | Severe pain or discomfort | 1.4 | 0.594 | 5.8 | 1.667 | 3.4 | 0.830 |  | Extreme pain or discomfort | 0.2 | 0.167 | 0.6 | 0.418 | 0.4 | 0.209 |
|  | Extreme pain or discomfort | 0.1 | 0.098 | 0.1 | 0.132 | 0.1 | 0.079 |  |  |  |  |  |  |  |  |
| Anxiety/ Depression | Not anxious or depressed | 79.8 | 2.622 | 69.7 | 4.327 | 75.1 | 2.454 | Anxiety/ Depression | Not anxious or depressed | 84.4 | 3.015 | 78.6 | 4.143 | 81.7 | 2.403 |
|  | Slightly anxious or depressed | 15.9 | 2.414 | 17.1 | 2.036 | 16.4 | 1.637 |  |  |  |  |  |  |  |  |
|  | Moderately anxious or depressed | 3.7 | 0.682 | 7.8 | 1.988 | 5.6 | 0.94 |  | Moderately anxious or depressed | 12.1 | 1.391 | 19.7 | 3.638 | 15.6 | 1.851 |
|  | Severely anxious or depressed | 0.6 | 0.318 | 5.0 | 1.512 | 2.6 | 0.718 |  | Extremely anxious or depressed | 3.5 | 3.109 | 1.7 | 0.879 | 2.7 | 1.719 |
|  | Extremely anxious or depressed | 0.1 | 0.069 | 0.4 | 0.337 | 0.2 | 0.155 |  |  |  |  |  |  |  |  |

SE, standard error; %, percentage

**Supplementary table 8 - Most frequently reported EQ-5D-5L health states and their mean index values and EQ VAS scores. Weighted sample.**

| **Health states** | **n** | **Percentage (%)** | **Cumulative %** | **Mean index value** | **Mean EQVAS (SE)** |
| --- | --- | --- | --- | --- | --- |
| 11111 | 829 | 50.3 | 50.3 | 1.000 | 90.9 (0.566) |
| 11112 | 72 | 4.4 | 54.7 | 0.962 | 88.0 (1.008) |
| 11121 | 134 | 8.2 | 62.8 | 0.947 | 84.1 (0.894) |
| 11122 | 43 | 2.6 | 65.5 | 0.909 | 83.8 (1.975) |
| 11221 | 21 | 1.3 | 66.7 | 0.908 | 78.5 (1.872) |
| 11222 | 17 | 1.0 | 67.8 | 0.870 | 77.3 (3.371) |
| 21111 | 20 | 1.2 | 69.0 | 0.961 | 80.3 (1.625) |
| 21121 | 37 | 2.2 | 71.2 | 0.908 | 79.3 (1.870) |
| 21221 | 16 | 1.0 | 72.2 | 0.869 | 67.9 (2.335) |

**SE**, standard error; **%**, percentage;

**Supplementary table 9 - Most frequently reported EQ-5D-3L health states and their mean index values and EQ VAS scores. Weighted sample.**

| **Health states** | **n** | **%** | **Cumulative %** | **Mean index value** | **Mean EQVAS (SE)** |
| --- | --- | --- | --- | --- | --- |
| 11111 | 1010 | 61.2 | 61.2 | 1.000 | 89.2 (0.657) |
| 11112 | 58 | 3.5 | 64.8 | 0.914 | 85.2 (1.627) |
| 11113 | 23 | 1.4 | 66.2 | 0.762 | 85.5 (8.729) |
| 11121 | 108 | 6.6 | 72.7 | 0.896 | 79.2 (1.381) |
| 11122 | 53 | 3.2 | 75.9 | 0.842 | 74.3 (1.367) |
| 11221 | 23 | 1.4 | 77.3 | 0.852 | 72.7 (3.617) |
| 21111 | 19 | 1.1 | 78.5 | 0.930 | 78.8 (1.918) |
| 21121 | 39 | 2.3 | 80.8 | 0.858 | 70.4 (2.809) |
| 21211 | 17 | 1.0 | 81.8 | 0.886 | 65.9 (1.451) |
| 21221 | 39 | 2.4 | 84.2 | 0.814 | 56.8 (3.751) |
| 21222 | 18 | 1.1 | 85.3 | 0.760 | 63.9 (5.296) |
| 22221 | 44 | 2.6 | 87.9 | 0.774 | 53.1 (3.297) |
| 22222 | 68 | 4.1 | 92.0 | 0.720 | 54.3 (5.372) |

**SE**, standard error; **%**, percentage;
